# Chronic liquid fructose supplementation does not cause liver tumorigenesis but elicits clear sex differences in the metabolic response in Sprague–Dawley rats

**DOI:** 10.29219/fnr.v65.7670

**Published:** 2021-09-22

**Authors:** Nuria Roglans, Miguel Baena, Gemma Sangüesa, Ana Magdalena Velázquez, Christian Griñán-Ferré, Mercè Pallàs, Rosa María Sánchez, Marta Alegret, Juan Carlos Laguna

**Affiliations:** 1 Department of Pharmacology, Toxicology and Therapeutic Chemistry, School of Pharmacy and Food Science, University of Barcelona, Barcelona, Spain; 2 Institute of Biomedicine, University of Barcelona, Barcelona, Spain; 3 Centro de Investigación Biomédica en Red de Fisiopatología de la Obesidad y Nutrición (CIBERObn), Madrid, Spain; 4 Institute of Neuroscience (UBNeuro), University of Barcelona, Barcelona, Spain

**Keywords:** diethyl nitrosamine, hepatocellular cancer, metabolic syndrome, sugar-sweetened beverages, sugary drinks

## Abstract

**Background:**

Non-alcoholic fatty liver disease (NAFLD) has increased over the last decades and may evolve into hepatocellular carcinoma (HCC). As HCC is challenging to treat, knowledge on the modifiable risk factors for NAFLD/HCC (e.g. hyper caloric diets rich in fructose) is essential.

**Objective and design:**

We used a model of diethyl nitrosamine-induced hepatocarcinogenesis to investigate the liver cancer-promoting effects of a diet supplemented with 10% liquid fructose, administered to male and female rats for 11 months. A subset of the fructose-supplemented rats received resveratrol (RVT) in the last 4 months of treatment.

**Results and discussion:**

Rat livers showed no *de visu* or histological evidence of liver tumorigenesis. However, we observed metabolic abnormalities that could be related to cancer development mainly in the female fructose-supplemented rats, such as increases in weight, adiposity and hepatic triglyceride levels, as well as hyperglycaemia, hyperuricemia, hyperleptinemia and a reduced insulin sensitivity index, which were partially reversed by RVT. Therefore, we performed a targeted analysis of 84 cancer-related genes in the female liver samples, which revealed expression changes associated with cancer-related pathways. Analysis of individual genes indicated that some changes increased the risk of hepatocarcinogenesis (*Sfrp2*, *Ccl5*, *Socs3*, and *Gstp1*), while others exerted a protective/preventive effect (*Bcl2* and *Cdh1*).

**Conclusion:**

Our data clearly demonstrate that chronic fructose supplementation, as the sole dietary intervention, does not cause HCC development in rats.

## Popular scientific summary

- Fatty liver may evolve into hepatocellular carcinoma, a liver tumour with no real effective therapy.
- Hyper caloric diets, such as those rich in fructose-sweetened beverages, are a modifiable risk factor for fatty liver and hepatocellular carcinoma.
- We tested whether an 11-month course of liquid fructose supplementation in rats induced liver tumorigenesis.
- There were no visual, histological or gene expression results confirming that liquid fructose supplementation, as the sole dietary factor, promotes liver tumorigenesis in rats.

The incidence of non-alcoholic fatty liver disease (NAFLD), a spectrum of pathologies ranging from simple steatosis to steatohepatitis, with or without fibrosis, has increased over the last decades (1). In a subset of patients, NAFLD may evolve into cirrhosis and then into hepatocellular carcinoma (HCC) (1). However, recent reports suggest that HCC may also develop in NAFLD patients without cirrhosis, and that even simple steatosis may be a risk factor for this type of cancer (2). As HCC is challenging to treat (3), a preventive strategy may be the best approach. Therefore, knowledge of the risk factors leading to NAFLD and HCC is essential. The consumption of hyper caloric diets rich in simple sugars, such as fructose, has a prominent role among these risk factors (4–6). The influence of dietary carbohydrates on HCC risk has been examined in several observational studies. Two case–control studies showed that the consumption of diets with a high glycemic index was associated with a higher risk of HCC, with the reduction of the dietary glycemic load lowering the risk or delaying the development of this type of cancer (7, 8).

Moreover, the European Prospective Investigation into Cancer and Nutrition study, which examined a cohort of 477,000 individuals, showed a positive correlation between total sugar intake and total soft drink consumption (sugar- and artificially sweetened beverages) and HCC risk (9, 10). Although fructose consumption, especially from liquid sources, is a direct dietary factor associated with NAFLD (11), the role of individual sugars as dietary risk factors for HCC is less clear. Some studies suggest that fructose consumption, despite increasing all-cause and cardiovascular mortality, is not associated with cancer mortality (12).

A few studies in rodent models have also been conducted to decipher the relationship between excessive fructose consumption and HCC, and the mechanisms involved (13–18). All of these, except for that of Kumamoto et al. (13), used mice as an experimental model. The rat is a good model for the study of fructose metabolism because, unlike other animal species like mice, but similar to humans, it does not transform ingested fructose into glucose due to the lack of intestinal glucose-6-phosphatase (19). Unfortunately, in the study of Kumamoto et al. (13), fructose supplementation was administered to rats for only 8 weeks in the form of a solid diet (66% of fructose). In the human diet, the main source of fructose is beverages sweetened with high fructose corn syrup or sucrose. It has been shown that the consumption of fructose in liquid form is more detrimental than when ingested in solid foods (20). Furthermore, 2 month is a short-time frame when compared with the usual pattern of sugar consumption in humans (21).

To overcome these shortcomings, we used a model of hepatocarcinogenesis in rats, which involved a single inducer intraperitoneal injection of diethyl nitrosamine (DEN) (22). Using this model, we investigated the promoter effect of a diet supplemented with 10% liquid fructose in the drinking water for 11 months in male and female rats, roughly equivalent to 20–30 years of human life (21). Resveratrol (RVT) has shown in different experimental animal models’ beneficial effects on lifespan, energy metabolism, inflammatory processes, cardiovascular risk factors and neurodegenerative diseases (23); moreover, RVT treatment has shown to prevent liver steatosis and cancer development in another model of liver carcinogenesis, such as the hepatitis B virus X protein mouse model (24). For this reason, a subset of the fructose-supplemented rats received RVT in the last 4 months of treatment in order to assess a possible beneficial effect of RVT on fructose-induced liver carcinogenicity. Despite no *de visu* or histological evidence of liver tumorigenesis, we observed metabolic abnormalities only in the female fructose-supplemented rats, which were partially reversed by RVT treatment. Targeted real-time polymerase chain reaction (RT-PCR) array analysis of 84 cancer-related genes in the female liver samples revealed changes in the expression of genes associated with cancer-related pathways in the livers of fructose-supplemented rats. However, individual gene expression changes indicated that some changes increased the risk of developing liver tumours, while others exerted a protective/preventive effect.

## Material and methods

### Animals and treatments

Male and female Sprague–Dawley rats (Charles River, Barcelona, Spain) were maintained with water and food ad libitum at constant humidity and temperature, with a light/dark cycle of 12 h and were mated to produce litters. Offspring male and female rats at the age of 16 days received a single intraperitoneal injection of DEN (5 mg/kg), and 2 weeks later, they were randomly assigned to a control group (CT) and a fructose-supplemented group (FRC) (10 and 20 rats of each sex per group, respectively). Fructose was supplied as a 10% (w/v) solution in drinking water for 11 months. Although this concentration of fructose almost doubles that presented in commercial beverages (25), it is frequently used as the standard dose of liquid fructose supplementation to rats (26), in order to compensate for the disproportionally short-life span of rats in comparison to humans. A subset of 20 fructose-supplemented rats (10 males and 10 females, F+RVT) received trans-RVT (2.2 and 3 mg RVT/kg of diet, respectively) for the last 4 months of treatment, which amounted to a daily RVT dose of 72 ± 7 and 80 ± 18 mg/kg of rat weight, respectively (*P* = 0.408). Throughout the treatment, solid food and liquid consumption were controlled weekly, and body weight was assessed once a month. Additional groups of male and female rats (*n* = 6/group) were used as no-DEN controls (NoDEN-CT), and their food and liquid consumption throughout the treatment was recorded and did not differ from DEN-treated controls (CT).

All procedures were conducted in accordance with the guidelines established by the University of Barcelona’s Bioethics Committee (Autonomous Government of Catalonia’s Act 5/1995, July 21) and the University of Barcelona’s Animal Experimentation Ethics Committee approval no. 8183).

### Sample Obtention

At the end of the treatment, after a 2-h fasting period, triglycerides and glucose levels were measured in blood samples obtained from the tail vein using an Accutrend^®^ Plus system glucometer (Cobas, Roche Farma, Barcelona, Spain). Then, rats were anesthetised with ketamine/xylazine (90:10 mg/kg body weight), and blood was collected by intracardiac puncture.

Plasma was prepared by centrifugation at 1,000 × *g* for 10 min at room temperature using micro tubes (Sarstedt AG & Co., Nümbrecht, Germany). The liver and visceral white adipose tissues (vWAT, including retroperitoneal, perirenal, and perigonadal adipose tissues) were obtained and weighed. All the samples were frozen in liquid nitrogen and stored at −80°C until needed.

### Plasma lipids and hormones

Plasma adiponectin, insulin, and leptin concentrations were determined using specific enzyme-linked immunosorbent assay kits (EZRADP-62K, EZRMI-13K, and EZRL-83K, respectively) from Millipore (Billerica, MA, USA). Insulin growth factor 1 (IGF1) determination was carried out using enzymatic kit (ab213902, Abcam, Cambridge, UK). Non-esterified fatty acid (NEFA) colorimetric kit was obtained from BiooScientific (Austin, TX, USA) and Uric acid kit from SpinReact (Girona, Spain) (Ref. no. 1001010). Plasma samples were assayed in duplicate.

### Liver triglyceride content

Liver triglycerides were extracted with chloroform/methanol (2/1 v/v) and measured as described elsewhere (27) using a triglyceride colorimetric kit (Ref: 41030) from Spinreact (Girona, Spain).

### RNA isolation and qPCR arrays

Total RNA was isolated from liver by using the Trizol^®^ reagent (Invitrogen, Carlsbad, CA, USA) in accordance with the manufacturer’s instructions. NucleoSpin RNA Clean-Up (Macherey-Nagel, Düren, Germany) was used for RNA purification. RNA concentration was determined by measuring absorbance at 260 nm, and the absorbance ratio 260/280 nm was used to analyse the RNA quality.

One microgram RNA was reverse transcribed to cDNA using the StaRT Reverse Transcriptase kit (StaRT-50). The rat Liver Cancer qPCR SignArrays^®^ was carried out in the StepOnePlus^TM^ Real-Time PCR System Thermal Cycling Block (Applied Biosystems, Foster City, CA) using SYBR Green Perfect Master Mix with ROX in 96 well-plates, following the manufacturer’s protocol. All these reagents were obtained from AnyGenes, Paris, France.

### Gene expression profile analysis and protein–protein interaction (PPI) network

To evaluate the gene expression profile, the data were initially examined by hierarchical clustering to determine changes among groups. We normalised using the SignArrays^®^ analysis tool from Any Genes, Paris, France. Fold changes of gene expression for heatmap were analysed using the statistical software BioVinci^®^. To investigate the molecular mechanisms, mRNAs that had fold changes of more than two in expression compared to the CT were selected, considered significant and used to construct the PPI network using the biological online database tool (Search Tool for the Retrieval of Interaction Genes, STRING, https://string-db.org) (28), to discriminate and predict the interaction among them. Furthermore, selected genes were chosen to be validated in an additional experiment.

### mRNAs validation by single real-time PCR

To validate the selected genes, we performed a RT-PCR using 100 μM of each specific primer and 10–20 ng of cDNA for each gene. mRNA expression was calculated using the recommended 2-^ΔΔCt^ method. The *TATA box-binding protein (tbp)* was used as a housekeeping gene to normalise the results. Table 1 summarises the sequences for selected genes used in this study. Each experiment was evaluated by three PCR reactions and repeated at least two times.

**Table 1:** SYBR Green Primers used in qPCR studies

Target	Forward primer (5’–3’)	Reverse primer (5’–3’)
***Bcl2***	TCTCATGCCAAGGGGGAAAC	TATCCCACTCGTAGCCCCTC
***Ccl5***	ATATGGCTCGGACACCACTC	GCGGTTCCTTCGAGTGACAA
***Cdh1***	TTGAGAATGAGGTCGGTGCC	CAGAATGCCCTCGTTGGTCT
***Cdkn2a***	GGTCGTACCCCGATACAGGT	TACCGCAAATACCGCACGAC
***Cflar***	ATTTTGTGGACCCCAAGGCA	ACTGGATCGAACGAGACACG
***E2f1***	ATTGAGCAGACCAAAGGGGG	AAGTGCTGTTAGAGCCCACC
***Gstp1***	GGGTCGCTCTTTAGGGCTTT	TTGCATCGAAGGTCCTCCAC
***Met***	AGTCCTACATTGATGTCCTGGGAG	ATGTAGGAGTGCAACCCAGAG
***Msh2***	GCTTCTATCCTCAGGTCGGC	AACCCAAAGCCGTCGTATGT
***Ptgs2***	TGAGTACCGCAAACGCTTCT	TCCTCCGAAGGTGCTAGGTT
***Socs1***	CCTTCGACTGCCTCTTCGAG	TTAAGAGGGATGCGTGCCAG
***Socs3***	CTTTACCACCGACGGAACCT	CCGTTGACAGTCTTCCGACA
***Srfp2***	GATCACCTCCGTGAAACGGT	CGGAAATGAGGTCGCAGAGT
***Tbp***	TGGGATTGTACCACAGCTCCA	CTCATGATGACTGCAGCAAACC

### GO and KEGG pathway analysis of mRNAs

To evaluate the mRNAs at the functional level, gene ontology (GO) enrichment analysis of molecular functions and Kyoto Encyclopaedia Genomes (KEGG) pathway analysis of the mRNAs were performed. In this study, the online tool for GO and KEGG analysis was Database for Annotation, Visualization and Integrated Discovery (DAVID, https://david.ncifcrf.gov/home.jsp) (29). Finally, the GO terms and KEGG pathways with the cut-off criteria (*P* < 0.05) were selected as the enriched functionality of the mRNAs. The *P*-value was calculated using the right-sided hypergeometric test; GO categories with *P*-values adjustment <0.05 were considered statistically significant.

### Western blot analysis

Thirty micrograms of total protein from rat livers were obtained and subjected to 10% SDS-polyacrylamide gel electrophoresis basically as described previously (30). Proteins were then transferred to Immobilon polyvinylidene difluoride transfer membranes (Millipore, Billerica, MA, USA), blocked for 1 h at room temperature with a 5% non-fat milk solution in 0.1% Tween-20-Tris-buffered saline (TBS), and incubated overnight at 4°C with the primary antibody raised against V-akt murine thymoma viral oncogene homolog-2 (AKT), pAKT, B-cell lymphoma 2 (BCL2) (dilution 1:1,000) (#9272, #4060, #3869, respectively; Cell Signalling, Leiden, Netherlands) and anti-glutathione s-transferase Pi1 (GSTP1) (dilution 1:500; NBP2-16756 from Novus Biologicals LLC., CO, USA). Detection was performed with the Thermo Scientific™ Pierce™ ECL Western blotting Substrate (Ref. no. 10455145, Thermo Scientific™, Madrid, Spain). To confirm the uniformity of protein loading, the blots were incubated with the β-ACTIN antibody (Sigma-Aldrich, St. Louis, MO) as a control.

### Histological studies

Paraffin-embedded liver sections were stained with haematoxylin–eosin to assess the degree of necrosis with Masson’s Trichrome for fibrosis analysis. A pathologist blinded to the treatment groups performed the histological analysis at Banc de Tumors, Hospital Clínic-IDIBAPS, Barcelona, Spain). Necrosis was scored as 0 (absent), 1 (<1%), 2 (<5%), 3 (<10%) or 4 (≥10%). Fibrosis was scored as 0 (absent), 1 (portal fibrosis without septa), 2 (1 plus some septa), 3 (abundant septa without cirrhosis) and 4 (cirrhosis).

### Statistics

The results are expressed as mean of *n* values ± standard deviation. Significant differences were established by Student’s *t*-test (female DEN and No-DEN data), two-way ANOVA (male and female zoometric and biochemical data), and by one-way ANOVA, followed by Sidak’s post-test analysis (mRNA WB and mRNA data from female rats). When the number of samples was too small or variance was not homogeneous, a non-parametric test was performed. The level of statistical significance was set at *P* < 0.05. The statistical analysis was conducted using the GraphPad Prism ver. 8 statistical software. Fold changes of the expression profile of the 84 mRNAs were calculated by using SignArrays^®^ data analysis. Candidate genes were selected when the differences were higher than 2-fold compared with the CT.

## Results

### Caloric intake and zoometrical parameters

Caloric intake and zoometrical parameters of the different groups (CT, FRC and FRC+RVT), segregated by sex, are shown in Table 2. Several trends can be observed from the data presented in Table 2. First, male rats had a significant higher final body weight, femur length, liver weight, ratio of liver weight to femur length, solid food intake and total calorie intake than female rats, pointing to a clear and expected sexual dimorphism for these parameters. Second, while liquid fructose supplementation significantly reduced solid food consumption and increased liquid fructose as well as total calorie intake in both sexes to a similar extent, only female rats showed a significant increase in body weight (×1.2) and vWAT weight (×1.7). Liver weight was increased in both sexes (×1.2 and ×1.4 for male and female rats, respectively). Third, male rats that received RVT and fructose showed higher body weight and total caloric intake than fructose-fed males.

**Table 2:** Caloric intake and zoometric parameters of Sprague–Dawley rats corresponding to the different study groups (CT, control; FRC, fructose; F+RVT, fructose plus resveratrol), segregated by rat sex

	Male rats			Female rats		
	CT	FRC	FRC + RVT	CT	FRC	FRC + RVT
Final body weight (g)	581 ± 46^a^	586 ± 53^a^	647 ± 76^a^*^#^	319 ± 16	397 ± 56*	358 ± 43
Femur length (cm)	4.3 ± 0.1^a^	4.6 ± 0.2^a^	4.7 ± 0.2^a^**	3.8 ± 0.6	3.9 ± 0.3	3.8 ± 0.3
vWAT weight (g)	11.5 ± 3.0	11.7 ± 3.5	14.6 ± 4.6	7.7 ± 1.9	13.4 ± 5.7*	10.7 ± 3.6
vWAT weight/femur length (g/cm)	2.6 ± 0.6	2.6 ± 0.8	3.1 ± 0.9	2.1 ± 0.7	3.4 ± 1.5*	2.8 ± 0.9
Liver weight (g)	17.1 ± 1.5^a^	20.5 ± 2.2^a^*	21.9 ± 3.8^a^***	9.4 ± 0.9	13.4 ± 2.8*	12.4 ± 1.8*
Liver weight/femur length (g/cm)	3.8 ± 0.6^a^	4.3 ± 0.8^a^	4.6 ± 0.7^a^**	2.5 ± 0.3	3.3 ± 0.8**	3.3 ± 0.3*
AUC liquid intake (mL/2 rats × 11 months)	2,3624 ± 3,666	80,651 ± 24,644*	75,624 ± 15,398*	21,600 ± 2,744	72,676 ± 12,776**	7,5218 ± 14,980**
AUC solid food intake (g/2 rats × 11 months)	14,970 ± 821	8,869 ± 1,618^b^***	10,621 ± 1,045^a^***	11,008 ± 827	6,808 ± 573***	6,392 ± 809***
AUC Kcal from fructose (2 rats × 11 months)	0	32,683±10,051	34,465 ±6,153	0	29,444 ± 5,200	30,433 ± 3,456
AUC total Kcal (2 rats × 11 months)	42,821 ± 2,242^a^	58,963 ± 5,567^c^***	62,144 ± 6,385^a^***^#^	32,495 ± 2,426	49,601 ± 4,080***	49,356 ± 4,673***

Effect of treatments on body weight, tissue weight, liquid and solid intake and total kcal consumed. Each value represents mean ± SD (n = 6–10). Different letters and symbols indicate statistical differences, established by two-way ANOVA test:

a *P* < 0.001,

b *P* < 0.01,

c *P* < 0.05 versus female rats;

*** *P* < 0.001,

** *P* < 0.01,

* *P* < 0.05 versus its respective sex control;

# *P* < 0.05 versus FRC group of the same sex.

**Fig. 1:**
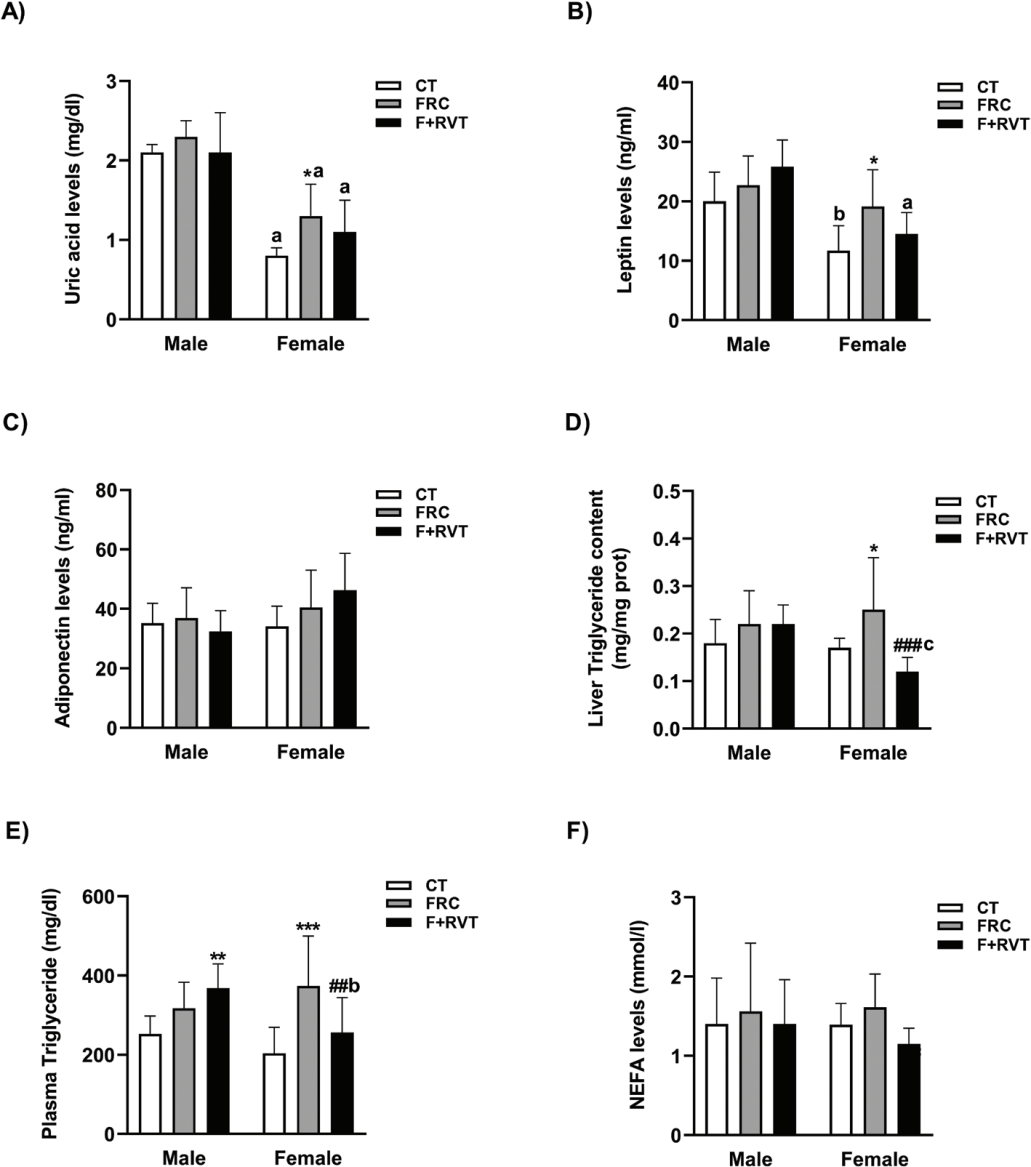
Biochemical parameters of Sprague–Dawley rats corresponding to the different study groups (CT, control; FRC, fructose; F+RVT, fructose plus resveratrol), segregated by rat sex. Plasma uric acid (A), leptin (B), adiponectin (C), triglyceride (E), non-esterified fatty acids (NEFA) (F) and liver triglyceride (D) concentrations. ^a^*P* < 0.001, ^b^*P* < 0.01 versus the corresponding male rat group; ^***^P < 0.001, **P < 0.01, *P < 0.05 versus its respective sex control; ^#^*P* < 0.05, ^##^*P* < 0.01, ^###^*P* < 0.001 versus fructose of the same sex.

As shown in Fig. 1, the lack of significant changes induced by liquid fructose supplementation in male rats was even more striking when looking at the biochemical parameters. Except for plasma uric acid and leptin levels (Fig. 1A and B), which were higher in control male than female rats (×2.6 and ×1.4, respectively), there was no significant difference between the control male and female rats in these parameters. However, fructose supplementation significantly increased plasma uric acid levels (×1.6), leptin levels (×1.6) as well as plasma triglyceride concentrations (×1.9) only in the female rats (Fig. 1A, D and E). RVT treatment significantly reduced liver and plasma triglyceride levels (×0.5 and ×0.7, respectively), with respect to fructose-supplemented female rats (Fig. 1D and E), also producing a non-significant reduction in all the remaining biochemical parameters assessed, except for adiponectin (Fig. 1C). RVT treatment showed no significant effect in male rats.

**Fig. 2:**
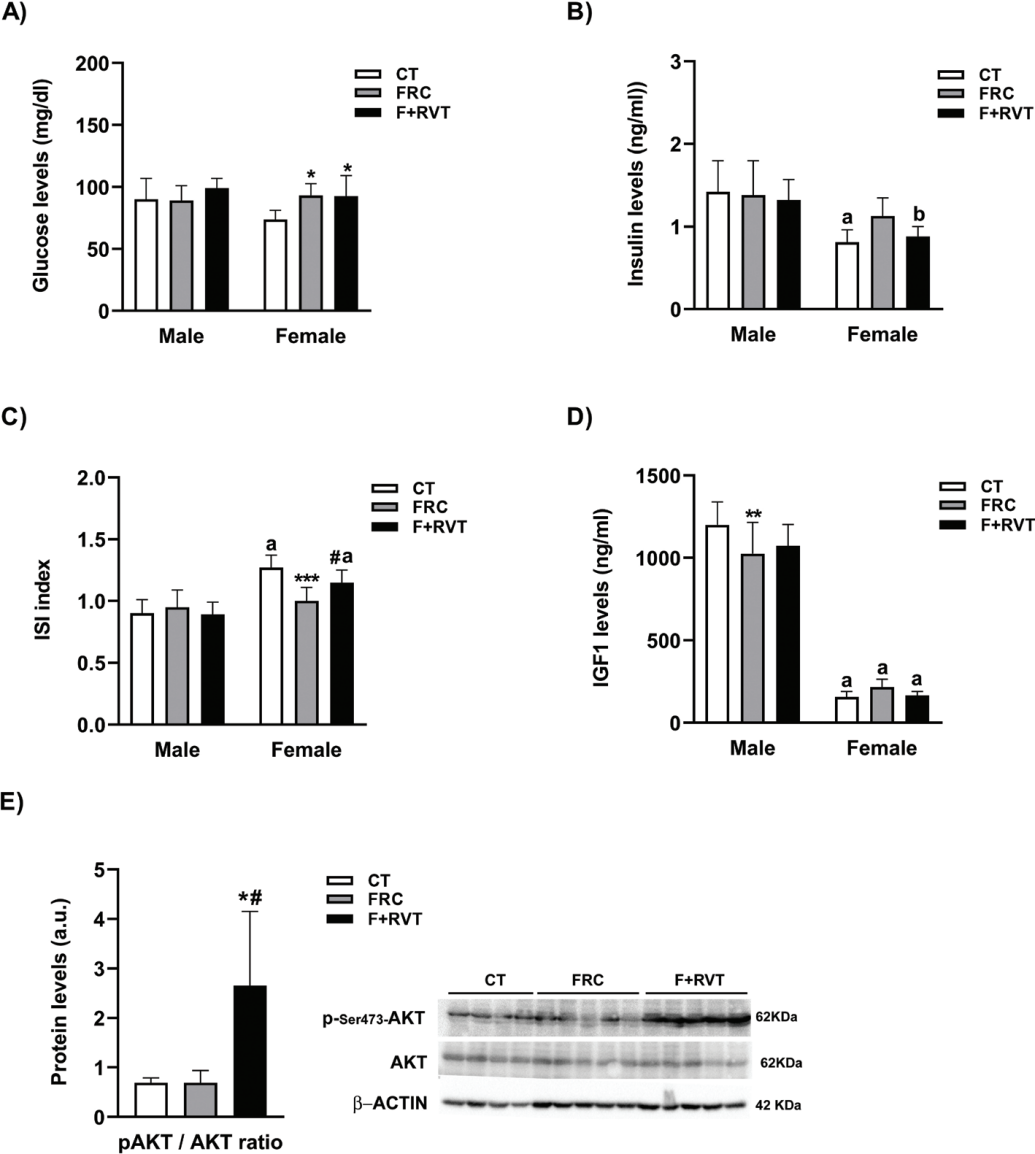
Biochemical parameters related to glycemia control of Sprague–Dawley rats corresponding to the different study groups (CT, control; FRC, fructose; F+RVT, fructose plus resveratrol), segregated by rat sex. Plasma glucose (A), insulin (B) and insulin growth factor 1 (IGF1) (D) concentrations, as well as insulin sensitivity index (ISI) values (C). (E) Bar plots showing the ratio of liver phosphor to total V-akt murine thymoma viral oncogene homolog-2 (Akt) expression in female rat livers (mean ± SD of four to five different liver samples). At the right side of the (E), actual WB bands corresponding to the three different study groups are shown. ^a^*P* < 0.001, ^b^*P* < 0.01 versus the corresponding male rat group; ***P < 0.001, **P < 0.01, *P < 0.05 versus its respective sex control; ^#^*P* < 0.05 versus fructose of the same sex.

Figure 2 shows the results for the parameters associated with glycaemic control. Control male rats had higher insulin concentrations than control female rats (×1.7) and presented a lower insulin sensitivity index (ISI, ×0.6), compared to control female rats. ISI was determined as described in (31). They also showed a marked difference in the plasma levels of IGF1 (×7.6). However, the huge calorie burden imposed by liquid fructose supplementation (increase of ×1.4 and ×1.5 in total calories for male and female rats, respectively; see Table 2), only worsened glycaemic control in the female rats. While the parameters related to glycaemic control did not change in the male rats, plasma glucose (×1.3) concentrations were increased in the female rats after liquid fructose supplementation. Furthermore, liquid fructose supplementation reduced the ISI (×0.8) only in the female rats, and this change was reverted by RVT treatment. Interestingly, changes in the expression of phosphorylated (Ser473) AKT2 paralleled changes in insulin sensitivity in the female rats.

### Liver histology

**Fig. 3:**
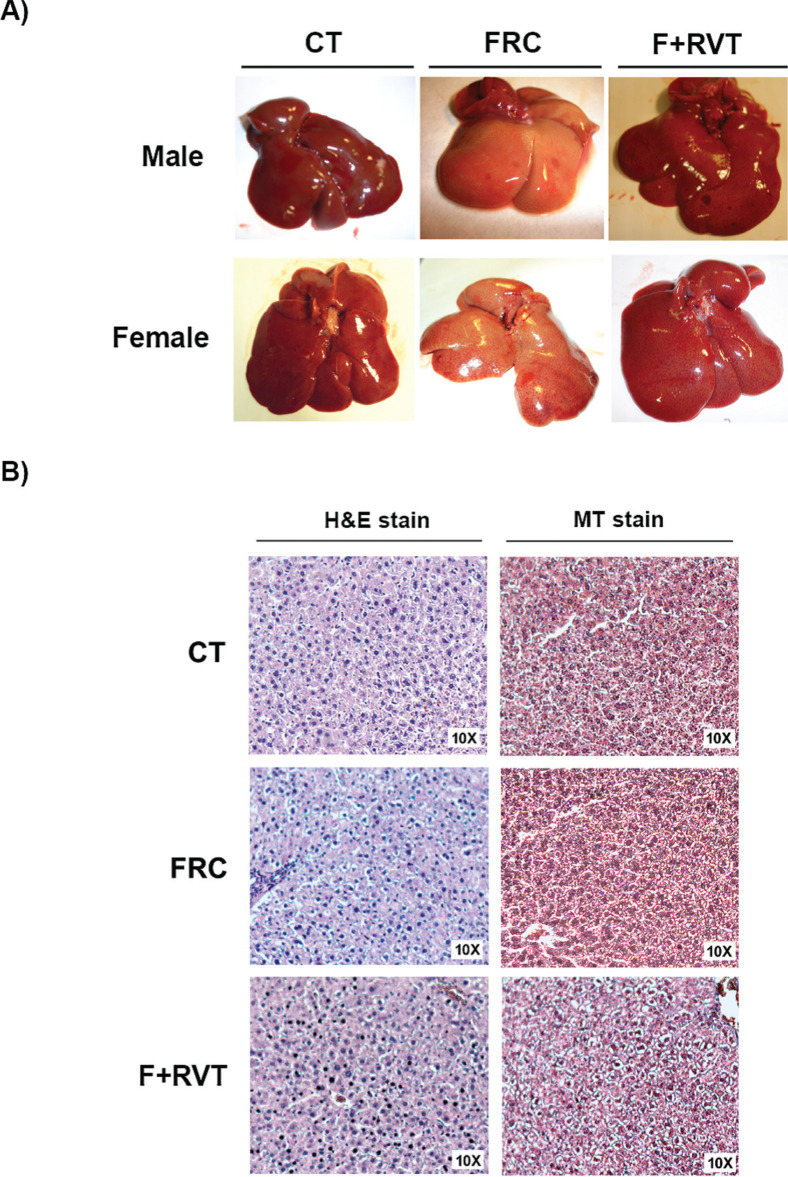
*De visu* (CT, control; FRC, fructose; F+RVT, fructose plus resveratrol) and histological analysis of livers from Sprague–Dawley rats corresponding to the different study groups. (A) Representative pictures of livers from male and female rats and (B) haematoxylin–eosin (10×) and Trichromic Masson (10×) representative stained liver samples of Sprague–Dawley female rats.

No visual morphological changes in the liver external architecture that could be attributed to a developing liver tumour were detected in any of the livers from the male and female (Fig. 3A) rats included in the study. Furthermore, analysis of histological scores for necrosis and fibrosis (Fig. 3B) using haematoxylin–eosin and Trichromic Masson stained liver samples, respectively, did not show any significant differences among the groups (0 score for necrosis and fibrosis in all samples).

### Effect of DEN administration on zoometrical and biochemical parameters in control female Sprague–Dawley rats

Despite the lack of *de visu* and histological evidence of liver tumorigenesis, liquid fructose supplementation worsened most of the zoometrical and biochemical parameters evaluated, specifically in female rats (see Table 2 and Fig. 1), with these changes partially reverted by RVT. Some of these alterations could be related to mechanisms involved in the initial phases of hepatocarcinogenesis, before the appearance of hepatic tumours. Thus, we were interested in determining the possible changes in molecular pathways associated with cancer and metabolism induced by liquid fructose supplementation, independently of DEN. Therefore, we previously ascertained that a single intraperitoneal injection of DEN (5 mg/kg) into 16-day-old female rats did not significantly alter any of these parameters. As can be seen in Table 3, when comparing the zoometrical and biochemical parameters of the female control rats treated with DEN (DEN Controls) with those of female rats maintained in the same conditions, but without receiving the injection of DEN (no DEN controls), there was no significant difference among any of the parameters.

**Table 3:** Zoometric and biochemical parameters of female control CT rats ± DEN administration

	Female rats	
	No DEN CT	DEN CT
Final body weight (g)	316 ± 35	319 ± 16
Femur length (cm)	3.8 ± 0.1	3.8 ± 0.3
vWAT weight (g)	5.6 ± 1.4	7.7 ± 1.9
vWAT weight/femur length (g/cm)	1.5 ± 0.4	2.1 ± 0.7
Liver weight (g)	8.5 ± 0.6	9.4 ± 0.9
Liver weight/femur length (g/cm)	3.8 ± 0.1	2.5 ± 0.3
*Plasma and biochemical analytes*		
Triglyceride (mg/dL)	179 ± 71	204 ± 65
Glucose (mg/dL)	78.3 ± 11.3	73.6 ± 7.5
Uric Acid (mg/dL)	0.8 ± 0.1	0.8 ± 0.1
NEFA (mmol/L)	2.09 ± 0.93	1.39 ± 0.27
Adiponectin (ng/mL)	32.0 ± 4.0	34.1 ± 6.8
Leptin (ng/mL)	14.4 ± 5.2	11.7 ± 4.2
Insulin (ng/mL)	0.93 ± 0.14	0.81 ± 0.15
ISI	1.18 ± 0.09	1.27 ± 0.10
IGF1 (ng/mL)	174 ± 28	157 ± 33
Hepatic Triglyceride (mg/mg prot)	0.18 ± 0.07	0.17 ± 0.02

Body and tissue weight, plasma and biochemical analytes in female control rats which received (DEN CT) or not received (no DEN CT) a single injection of diethyl nitrosamine (DEN) at the age of 16 days. Each value represents the mean ± SD (*n* = 6). Statistical analysis was performed using Student’s *t* test.

### Expression profile of genes associated with cancer and metabolic pathways is altered in the liver of female rats after fructose supplementation and reversed by RVT administration

**Fig. 4:**
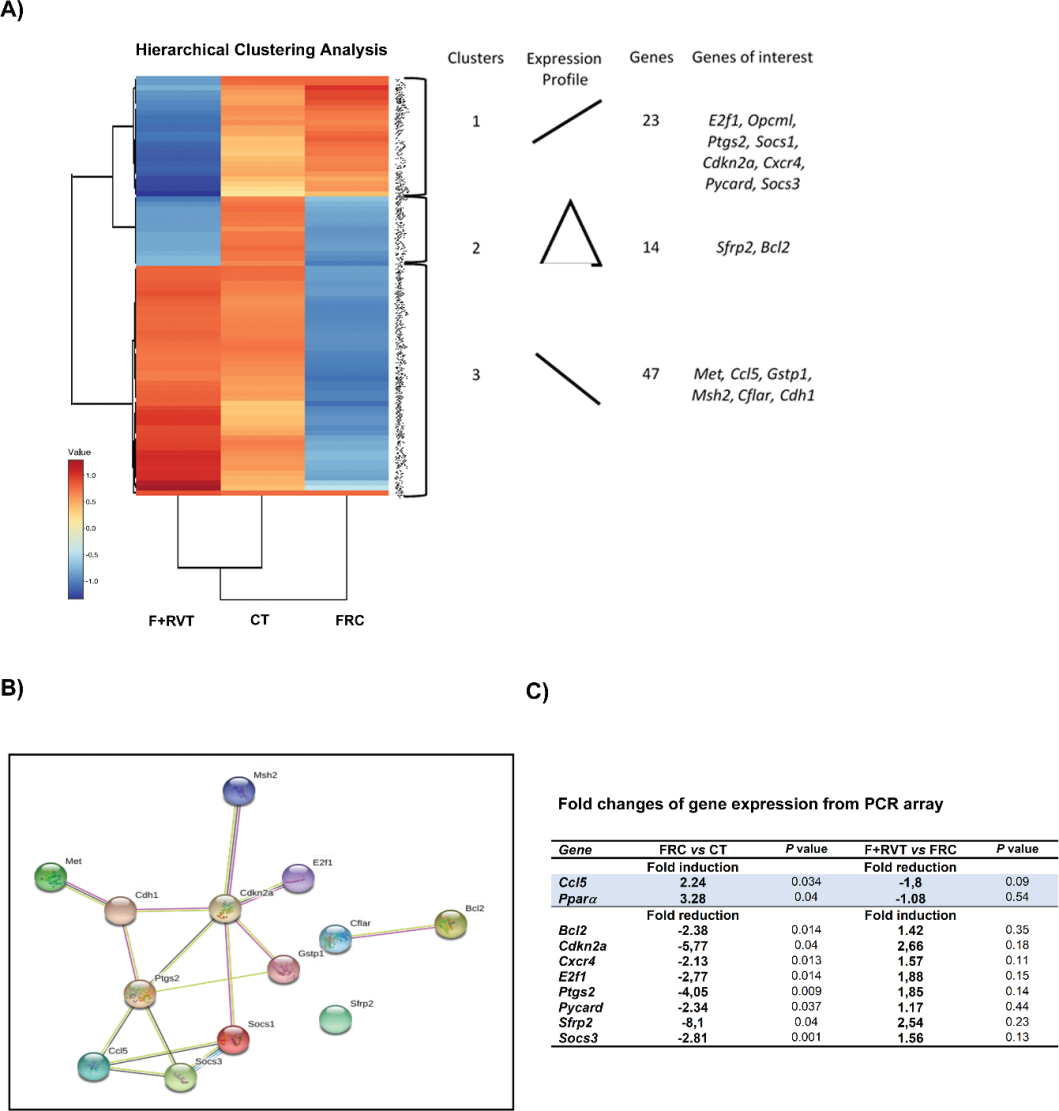
Targeted RT-PCR array of 84 genes associated with cancer and metabolic pathways in liver tissue samples of female Sprague–Dawley rats corresponding to the different study groups (CT, control; FRC, fructose; F+RVT, fructose plus resveratrol). (A) K-means hierarchical clustering analysis and heatmap of mRNA expression profile of rat liver from each group. STRING analysis of the relationship between selected genes. The network nodes represent the proteins of selected genes (B). Three groups (groups 1 to 3) with their relative expression changes depicted schematically under ‘Expression profile’. Each cluster contains different number of genes, and representative genes from each cluster are listed to the right. (B) Predicted protein–protein interactions (PPI) by STRING analysis of the relationship between selected genes. The network nodes represent the proteins of selected genes; GO terms and KEGG pathways with the cut-off criteria (*P* < 0.05) were selected as the enriched functionality of candidate genes. (C) Fold changes in gene expression from PCR array: *c-c motif chemokine ligand 5 (Ccl5)*, *peroxisome proliferator activated receptor alpha (Ppar*α*)*, *B-cell lymphoma 2 (Bcl2)*, *cyclin-dependent kinase inhibitor 2A (Cdkn2a)*, *C-X-C chemokine receptor 4 (Cxcr4)*, *e2f transcription factor 1 (e2f1), prostaglandin-endoperoxidase synthase 2 (Ptgs2)*, *apoptosis speck-like protein containing a CARD (Pycard)*, *secreted frizzled related protein 2 (Sfrp2)* and *suppressor of cytokine signalling 3 (Socs3)*. (C) Gene ontology (GO) enrichment analysis of molecular functions. (D) Kyoto encyclopedia genomes (KEGG) pathway analysis of the mRNAs.

We used a targeted RT-PCR array to assess whether liquid fructose supplementation and/or RVT treatment altered the transcript levels of 84 genes associated with cancer and metabolic pathways in the liver tissue samples of female rats. The hierarchical clustering analysis provides a graphical representation of the fold change in expression for each group (Fig. 4A), clearly showing a differentiated pattern of transcript levels among the three experimental groups (CT, FRC, and FRC+RVT). We identified a group of 10 genes in the livers of fructose-supplemented rats that displayed a significant change of more than 2-fold in their expression (8 were downregulated and 2 upregulated) compared with control rats (Fig. 4C). Interestingly, the expression of most of these genes did not remain significantly elevated or decreased in the group that received RVT treatment compared to control rats. Moreover, when comparing the F+RVT and FRC groups, the expression of the downregulated genes showed an increase, although these changes were non-significant (Fig. 4C). Furthermore, we constructed a regulatory network based on the predicted protein–protein interactions (Fig. 4B). The enrichment analysis with candidate mRNAs confirmed that the altered mRNA levels found were likely to have an impact on pathways associated with cell death, apoptosis, inflammation and, consequently, cancer.

### Functional categories based on GO and KEGG pathways

To evaluate the functions of the genes showing changes in their expression, we performed a pathway enrichment analysis of the K-means-clustered groups using the GO database. The analysis revealed expected and also novel changes in GO pathways (Table 4). We identified different enrichment for each K-means group (Table 5). K-means group cluster 1 showed enrichment of molecular functions associated with protein-containing and protein binding (GO: 0044877 and GO: 0005515). K-means cluster 2 revealed enrichment of protein binding and death effector domain, and K-means cluster 3 showed unexpected changes in enzyme binding and BH3 domain (GO: 0019899, GO: 0051434; GO: 0005515 and GO: 0035877, respectively). Also, we observed different enrichment in biological processes depending on the K-means cluster group. For instance, cluster 1 showed enrichment associated with cell death and regulation of death, cluster 2 showed enrichment associated with negative regulation of cellular metabolic process and unexpected change in cell surface receptor signalling, and cluster 3 revealed enrichment with programmed cell death and unexpected change in tube morphogenesis (GO: 0008219, GO: 0010941; GO: 0031324, GO: 007166; GO: 0012501 and GO: 0035239, respectively), as well as the cell component enrichment was membrane raft (GO: 0045121) for cluster 1, catenin complex (GO: 0016342) for cluster 2 and CD95 death-inducing signalling complex (GO: 0031265) for cluster 3. Remarkably, all these pathways are mainly involved in cancer, apoptosis and the cell death process (Fig. 4A).

**Table 4:** Functional categories based on GO pathways for each cluster

	Term ID	Term name	*P*-value_adj_
Cluster 1 GO biological process			
GO:MF	GO:0044877	Protein-containing complex binding	5.952 × 10^-12^
GO:MF	GO:0005515	Protein binding	3.901 × 10^-11^
GO:BP	GO:0008219	Cell death	2.697 × 10^-20^
GO:BP	GO:0010941	Regulation of cell death	1.179 × 10^-19^
GO:CC	GO:0045121	Membrane raft	4.959 × 10^-9^
Cluster 2 GO biological process			
GO:MF	GO:0019899	Protein binding	3.734 × 10^-4^
GO:MF	GO:0051434	Death effector domain binding	7.184 × 10^-4^
GO:BP	GO:0031324	Negative regulation of metabolic process	6.273 × 10^-7^
GO:BP	GO:0007166	Cell surface receptor signaling pathway	6.303 × 10^-7^
GO:CC	GO:0016342	Catenin complex	1.419 × 10^-2^
Cluster 3 GO biological process			
GO:MF	GO:0005515	Enzyme binding	5.283 × 10^-4^
GO:MF	GO:0035877	BH3 domain binding	1.613 × 10^-3^
GO:BP	GO:0012501	Programmed cell death	1.560 × 10^-7^
GO:BP	GO:0035239	Tube morphogenesis	2.793 × 10^-7^
GO:CC	GO:0031265	CD95 death-inducing signaling complex	4.468 × 10^-4^

**Table 5:** Functional categories based on KEGG pathways for each cluster

	Term ID	Term name	*P*-value_adj_
Cluster 1 KEGG pathway			
KEGG	KEGG:05200	Pathways in cancer	2.854 × 10^-24^
KEGG	KEGG:05210	Colorectal cancer	1.551 × 10^-15^
KEGG	KEGG:05225	Hepatocellular carcinoma	2.722 × 10^-14^
KEGG	KEGG:04151	PI3K-Akt signaling pathway	1.671 × 10^-14^
KEGG	KEGG:05205	Proteoglycans in cancer	2.105 × 10^-13^
Cluster 2 KEGG pathway			
KEGG	KEGG:05210	Colorectal cancer	3.948 × 10^-6^
KEGG	KEGG:05200	Pathway in cancer	4.363 × 10^-6^
KEGG	KEGG:05145	Toxoplasmosis	1.494 × 10^-5^
KEGG	KEGG:04210	Apoptosis	3.147 × 10^-5^
KEGG	KEGG:05226	Gastric cancer	5.803 × 10^-5^
Cluster 3 KEGG pathway			
KEGG	KEGG:05200	Pathways in cancer	6.246 × 10^-11^
KEGG	KEGG:05163	Human cytomegalovirus infection	6.158 × 10^-7^
KEGG	KEGG:05219	Bladder cancer	8.833 × 10^-7^
KEGG	KEGG:05206	MicroRNAs in cancer	1.785 × 10^-6^
KEGG	KEGG:05167	Kaposi sarcoma herpesvirus infection	5.212 × 10^-6^

### Validation of selected mRNAs involved in cancer, apoptotic and inflammatory pathways

**Fig. 5:**
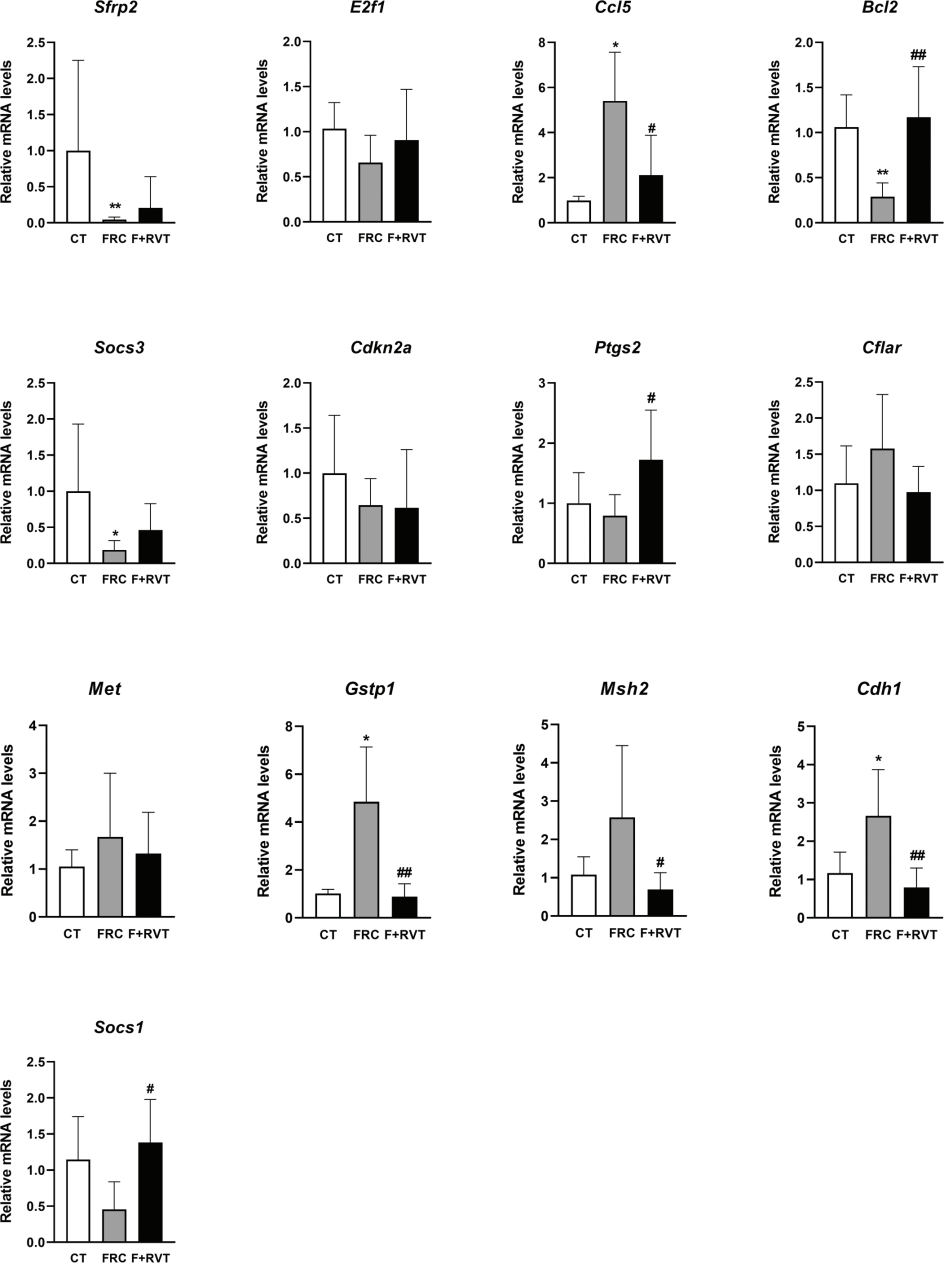
Validation of a representative subset of mRNAs involved in the main molecular pathways associated with metabolic response after chronic fructose in livers of female Sprague–Dawley rats. Relative liver expression of *secreted frizzled-related protein 2 (Sfrp2), e2f transcription factor 1 (E2f1), c-c motif chemokine ligand 5 (Ccl5), B-cell lymphoma 2 (Bcl2), suppressor of cytokine signalling 3 (Socs3), cyclin-dependent kinase inhibitor 2A (Cdkn2a), prostaglandin-endoperoxidase synthase 2 (Ptgs2), CASP8- and FADD-like apoptosis regulator (Cflar), met proto-oncogene receptor tyrosine kinase (Met), glutathione s-transferase Pi1 (Gstp1), mutS homolog 2 (Msh2), cadherin 1 (Cdh1) and suppressor of cytokine signalling 1 (Socs1)* in samples from the different study groups (CT, control; FRC, fructose; F+RVT, fructose plus resveratrol). Gene expression levels were determined by real-time PCR. Each bar represents the mean ± standard deviation (SD) (n = 6–8 for each group). *P < 0.05, **P < 0.01 versus control; ^#^*P* < 0.05, ^##^*P* < 0.01 versus FRC.

**Fig. 6:**
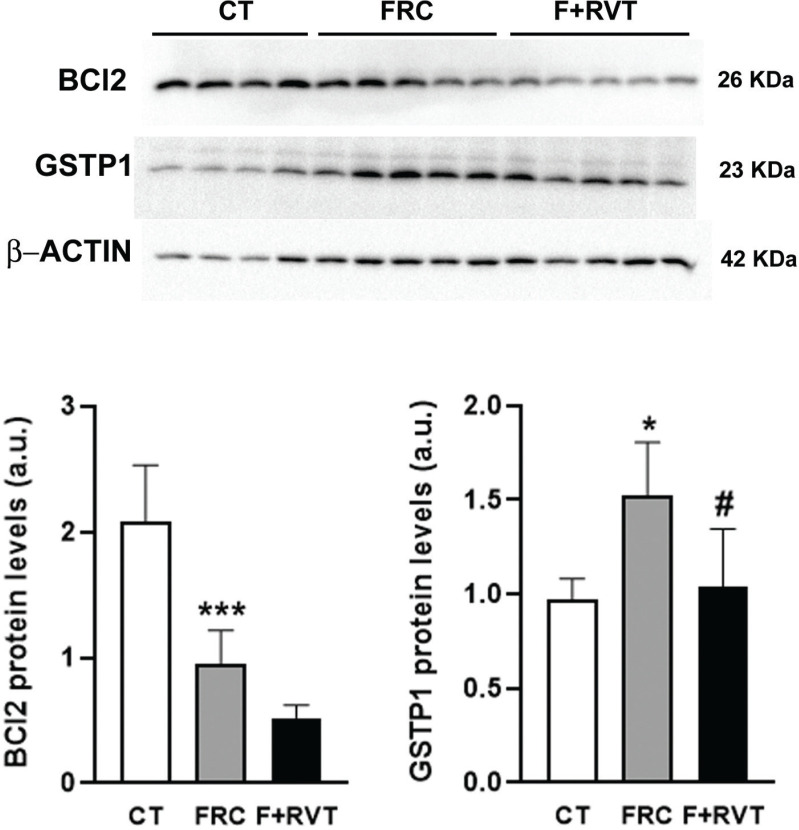
Western-blot (WB) analysis of B-cell lymphoma 2 (BCL2) and glutathione s-transferase Pi1 (GSTP1) proteins in female Sprague–Dawley rat liver samples from the different study groups (CT, control; FRC, fructose; F+RVT, fructose plus resveratrol). Bar plots show the relative WB band intensity as mean ± SD of 4–5 different liver samples. At the upper part of the figure, actual WB bands corresponding to the three different study groups are shown.

To validate the results of the gene profiling analysis, levels of several mRNAs selected according to the intensity of the observed changes and their biological relevance were measured by single real-time PCR in liver samples from each group. We assessed the expression of *secreted frizzled-related protein 2 (Sfrp2)*, *e2f transcription factor 1 (e2f1)*, *c-c motif chemokine ligand 5 (Ccl5)*, *Bcl2, suppressor of cytokine signalling 3 (Socs3), cyclin-dependent kinase inhibitor 2A (Cdkn2a) and prostaglandin-endoperoxide synthase 2 (Ptgs2)*, as well as other genes that followed a similar pattern of differential expression (*CASP8 and FADD like apoptosis regulator (Cflar), met proto-oncogene, receptor tyrosine kinase (Met), Gstp1, mutS homolog 2 (Msh2), cadherin 1 (Cdh1) and suppressor of cytokine signalling 1 (Socs1)*). Changes induced by fructose-supplementation were validated for *Sfrp2, Ccl5, Bcl2, Socs3, Gstp1* and *Cdh1*, while those significantly reverted by RVT treatment were validated for *Ccl5, Bcl2, Ptgs2, Gstp1, Msh2, Cdh1* and *Socs1*. Changes induced by fructose-supplementation and RVT treatment were validated only for *Bcl2, Ccl5, Gstp1* and *Cdh1* genes (see Fig. 5). Furthermore, we assessed if changes in mRNA levels translated into changes in protein levels for *Bcl2* and *Gstp1*. As shown in Fig. 6, changes in the protein levels of *Bcl2* and *Gstp1* followed a similar pattern to that of their corresponding mRNA levels.

## Discussion

In this work, we investigated the hepatic cancer-promoting effects of liquid fructose in male and female Sprague–Dawley rats using a single, initiator intraperitoneal dose of DEN (5 mg/kg), 2 weeks before starting promotion by fructose supplementation for 11 months. DEN administration *per se* did not cause hepatic tumours, as initially expected. We adapted the experimental protocol of Herranz et al. (22), who used an initiator dose of DEN of 5 mg/kg, and as a promoter, a high-fat diet regime maintained for 11 months to elicit liver tumorigenesis in mice. We used the same initiator dose of DEN as Kleinert et al., which is twice the dose that should be used in rats according to the ratio of the body surface areas of the two species (32). Thus, the lack of carcinogenic effect cannot be ascribed to an under dosage of DEN in the rats.

The fact that DEN did not act as a cancer initiator in our study precluded examining the effects of fructose as a cancer promoter. However, fructose may also exert carcinogenic effects *per se*. Recently, Ozawa et al. reported that chronic fructose feeding caused HCC in AIM^–/–^ mice in the absence of inflammation and fibrosis (17). This effect was attributed to a direct cytotoxic effect of fructose or its metabolites on hepatocytes, which caused an impairment in the liver and skeletal muscle insulin signalling cascade.

Despite the fact that both male and female Sprague–Dawley rats did not develop liver tumours after 11 months on liquid fructose supplementation, we focused on the metabolic abnormalities caused by fructose supplementation, as they could be related to a tumorigenesis-initiator effect of fructose that might be established even before the appearance of visible hepatic tumours.

Our first observation was that in our model, the response to liquid fructose-supplementation showed a marked difference between the sexes, with male rats practically unresponsive to the dietary challenge and female rats developing increased body weight with vWAT accretion, as well as metabolic disturbances when compared to the corresponding controls. This sex-related response was also observed with the RVT treatment provided in the last 4 months of fructose supplementation. RVT treatment in fructose-supplemented male rats was practically ineffective, but it partially or fully reverted most of the metabolic disturbances detected in the fructose-supplemented female rats.

Under standard laboratory husbandry, male and female Sprague–Dawley rats show an evident sex-related dimorphism in their growth curve, which start to plateau at around 15 weeks of age, with weighs of 375–575 and 225–350 g for males and females, respectively (33, 34). Our results indicated that after 11 months of a sedentary life with *ad libitum* access to food and liquid, male rats reached their maximum weight gain and metabolic flexibility. In contrast, female rats were still able to respond to a metabolic challenge, such as the increased caloric burden provided by the liquid fructose, as reflected by the significant gain in body weight and worsened metabolic parameters. Furthermore, these differences in metabolic flexibility/adaptability between the male and female rats were also observed in their responses to RVT, a well-known substance that can improve metabolic fitness in rodents (23). Only female rats showed improvements in their body weight and metabolic parameters in response to RVT treatment, at a daily dose that showed almost no effectiveness when provided in the male rats, probably due to the well-known metabolic effects of RVT associated with the activation of SIRT1 and AMPK signalling pathways (23). The lack of responsiveness in males to RVT administration is probably related to the higher metabolic activity in males than females, related to an inducing effect of androgen hormones, which could further reduce the already very low bioavailability of RVT.

The differences between the sexes were markedly evident in the parameters associated with glycaemic control. In rodents, males and females show a clear difference in glycaemic control (32), which was also observed in our present study. Male Sprague–Dawley rats showed higher plasma glucose and insulin concentrations and a lower ISI value than female rats, which were unchanged in response to liquid fructose supplementation. By contrast, the female rats supplemented with fructose showed an increase in their glycemia, reducing the ISI value. Again, regarding the RVT treatment, the male rats showed no changes in these parameters, while RVT-treated female rats showed a significant improvement in their ISI value.

Given the absence of metabolic derangements in male rats, we continued our data analysis exclusively on female rats. To investigate the metabolic and cancer-inducing effects of fructose independently of DEN, we previously ascertained that a single intraperitoneal injection of DEN into 16-day-old female rats did not significantly alter any of the metabolic parameters studied. Then, we used a targeted RT-PCR array to study the transcript levels of 84 genes associated with cancer and metabolic pathways in the liver of female fructose-supplemented rats.

Interestingly, our results revealed that fructose supplementation significantly modified the expression of genes associated with cell death, apoptosis and inflammatory pathways related to cancer development. Of the 10 genes in the livers of fructose-supplemented rats that displayed a significant change of more than 2-fold in their expression, we were able to validate these changes for six genes: *Sfrp2*, *Ccl5*, *Bcl2*, *Socs3*, *Gstp1* and *Cdh1*. We were also able to validate the changes for *Bcl2* and *Gstp1* at the protein level. Increased expression of *Ccl5* promotes tumour proliferation and metastasis (35), high expression of *Bcl2* inhibits apoptotic death, favouring neoplastic cells proliferation (36) and *Gstp1* is a marker of preneoplastic foci in liver tissue (37). On the contrary, decreased expression of *Sfrp2*, a physiological antagonist of Wnt signalling, promotes HCC progression (38); a reduction in *Socs3* expression, a marker of JAK-STAT signalling is related to an increased risk of cancer development (39); and, finally, lower levels of *Cdh1*-coded protein, E-cadherin, will facilitate the tissue-infiltration and metastasis of cancer cells (40). However, our results indicated a mixed situation, with some changes increasing the risk of developing liver tumours (*Sfrp2*, *Ccl5*, *Socs3* and *Gstp1*) and others exerting a protective/preventive effect against liver tumour development. This is the case, for example, of the fructose-related downregulation of Bcl2, a protein with antiapoptotic activity, which is a hallmark of many cancerous cells that present a deficit in apoptosis that facilitates tumour growth (36). In contrast, the increased E-cadherin protein expression (*Cdh1* gene) induced by fructose supplementation will potentiate cell-to-cell contact inhibition, reducing the mobility and growth of cancer cells (40).

## Conclusions

Our histology and gene expression data clearly indicate that chronic fructose supplementation, as the sole dietary intervention, does not cause HCC development in rats.
